# Extreme response style bias in burn survivors

**DOI:** 10.1371/journal.pone.0215898

**Published:** 2019-05-06

**Authors:** Pengsheng Ni, Molly Marino, Emily Dore, Lily Sonis, Colleen M. Ryan, Jeffrey C. Schneider, Alan M. Jette, Lewis E. Kazis

**Affiliations:** 1 Boston University School of Public Health, Department of Health Law, Policy, and Management, Boston, Massachusetts, United States of America; 2 Department of Surgery, Massachusetts General Hospital, Boston, Massachusetts, United States of America; 3 Harvard Medical School, Boston, Massachusetts, United States of America; 4 Shriners Hospitals for Children–Boston, Boston, Massachusetts, United States of America; 5 Department of Physical Medicine and Rehabilitation, Spaulding Rehabilitation Hospital, Boston, Massachusetts, United States of America; 6 MGH Institute of Health Professions, Boston, Massachusetts, United States of America; University of Notre Dame Australia, AUSTRALIA

## Abstract

This paper explores extreme response style to the Life Impact Burn Recovery Evaluation (LIBRE) Profile, a measure of social participation in burn survivors. We fit a Multidimensional Generalized Partial Credit Model (MGPCM) with a positive extreme response style (PERS) factor and compared this model with the original MGPCM, estimated the impact that PERS has on scores, and examined the personal characteristics that may result in an individual more likely to respond in a fashion that would inflate their true low scores. The average impact of the PERS, based upon the root mean squared bias, ranged from 0.27 to 0.50 of a standard deviation of the scale. Individuals who were older, had participated in a burn survivor support group, and had selected to self-administer the measure were less likely to have a high PERS bias that masks low scores. Future work can consider PERS when measuring the psychosocial impacts of burn injuries and other health conditions.

## Introduction

Clinicians increasingly use patient reported outcome measures (PROMs) to learn important information and optimize care.[1–3] However, PROMs can be sensitive to participants’ response styles, which may decrease the validity of the metric.[4] One type of bias is extreme response style (ERS). A person who tends towards an ERS is more likely to select either the most negative or the most positive response to a question compared to an individual who does not have this bias with the same ability/true score[5]. Individuals interpreting PROMs need to be aware of this phenomenon to account for this bias in results and better understand patient outcomes.

Previous studies have explored whether certain characteristics correlate with greater likelihood of choosing an extreme response, and have found that gender, age, educational levels, socio-economic status, and ethnicity affect response styles.[4,6,7] Studies also have shown that individual’s ERSs are consistent over several years.[8] Overall, little attention has been paid to developing methodologies that assess and control for response style effects in PROMs.

This paper explores the ERS of burn survivors who responded to the Life Impact Burn Recovery Evaluation (LIBRE) Profile, a validated measure of social participation in burn survivors. The development and examination of the psychometric properties for the LIBRE Profile are cited elsewhere.[9–11,12] Research has not yet examined ERS in burn survivors. With the unique experience of surviving a burn injury, and the long term effects of living as a burn survivor, this clinical group may display unique patterns of ERS. In this paper we ask if the sample shows extreme response bias, then examine if certain characteristics are associated with this response style and may inflate scores. Because previous studies have found varying results regarding characteristics associated with ERS,[4,7,13] we include a variety of demographic factors such as gender, education, and age, as well as clinical characteristics often used in the burn literature, such as time since burn injury and burn size. To improve the clinical utility of the LIBRE Profile, understanding ERS is essential to aid interpretation of scaled results.

## Materials and methods

### Sample and measure

The study included adult burn survivors 18 years or older, all subjects provided informed consent prior to participating in research study activities (S1 Appendix). Subjects who chose the mode of phone interviews provided verbal consent per the protocol, those who chose the self-administered survey checked off a box, acknowledging reading the informed consent and agreeing to participate in the study. The study was approved by the Boston University Medical Campus Institutional Review Board (Protocol H-32928). This analysis uses data from the sample of 601 burn survivors who provided information for the development of the initial LIBRE Profile[9]. The details regarding the population of burn survivors was previously described [9]. The LIBRE Profile currently comprises six unidimensional scales, three of which are core scales that pertain to all burn survivors in the sample: Relationships with Family & Friends, Social Interactions, and Social Activities. This analysis focuses on these three core scales with the full sample.

#### Building the IRT model to adjust the positive extreme response style (PERS) factor

We fit a Multidimensional Generalized Partial Credit Model (MGPCM) with a PERS factor which only impacts the highest category of each item. We chose to examine the positive extreme response category because few respondents selected the lowest category (we merged the lowest with the second lowest category for some items), and over half of the subjects selected the positive extreme response category in one third of the items. The formula for the item response theory (IRT) model is presented in **Formula 1**. Where: *k* = 1,2,…,*K*_*j*_ (*K*_*j*_ is the total number of categories for item j); *U*_*j*_ is participant’s response for an item *j*; *θ*_1_,*θ*_2_,*θ*_3_ are the substantive factors (Family & Friend, Social Interaction, and Social Activity) which correlated with each other; *a*_*j*,1_,*a*_*j*,2_,*a*_*j*,3_ are the discrimination parameters for substantive factors, assuming those values vary by item; *θ*_*PERS*_,*a*_*j*,*PERS*_ are the PERS bias factor (we assume no correlation between PERS bias and substantive factors) and corresponding discrimination parameter (we assume discrimination parameters vary by item); to identify the model, the substantive factors and PERS bias factor were assumed from standard normal distributions with mean = 0 and standard deviation = 1; *I*(*k* = *K*_*j*_) is the indicator function, if *k* = *K*_*j*_, then *I*(*k* = *K*_*j*_) = 1, otherwise *I*(*k* = *K*_*j*_) = 0; and finally *c*_*j*,*k*_ are the category difficulty parameters, and we assume *c*_*j*,1_ = 0.

**Formula 1.** IRT model that adjusts for PERS

$$P(U_{j}=k|\theta_{1},\theta_{2},\theta_{3},\theta_{PERS})=\frac{exp\;\left((k-1)(a_{j,1}\theta_{1}+a_{j,2}\theta_{2}+a_{j,3}\theta_{3})+I(k=K_{j})a_{j,PERS}\theta_{PERS}-c_{j,k}\right)}{\sum_{h=1}^{K_{j}}exp\;((h-1)(a_{j,1}\theta_{1}+a_{j,2}\theta_{2}+a_{j,3}\theta_{3})+I(h=K_{j})a_{j,PERS}\theta_{PERS}-c_{j,h})}$$ (1)

We assessed the model fit between this model with the original MGPCM which is not adjusted by PERS, by examining the information criteria (Akaike information criterion (AIC), Bayesian information criterion (BIC) and sample size adjusted BIC). AIC and BIC are information-theoretic methods for model selection. Both criteria include two components, one is the -2 times the log-likelihood of the estimated model which measure the model fit, another is the penalty term. The purpose of the penalty term is to avoid overfitting a model. The penalty term for AIC is 2m; the penalty term for BIC is m*ln(n). (m: number of parameters, n:sample size). In sample-size adjusted BIC, n is replaced by n*(n+2)/24. The information criteria favors the model with a large log-likelihood value but with few parameters, smaller values meaning better fit. We also examined the changes of substantive factor correlations in MGPCM with and without adjusting PERS.

Below, we give an example to explain how incorporating the PERS in the model can bias the probability of selecting a category. We present the category characteristic curves (CCCs) under three different PERS levels (the model without PERS bias (*θ*_*PERS*_ = 0), the model with positive PERS bias (*θ*_*PERS*_ = 1), and the one with negative PERS bias (*θ*_*PERS*_ = −1)). We assumed the item parameters as following: *a*_*j*,1_ = 1,*a*_*j*,2_ = *a*_*j*,3_ = 0,*a*_*j*,*PERS*_ = 1,*c*_*j*,2_ = −1,*c*_*j*,3_ = 0,*c*_*j*,4_ = 1.

**Fig 1:**
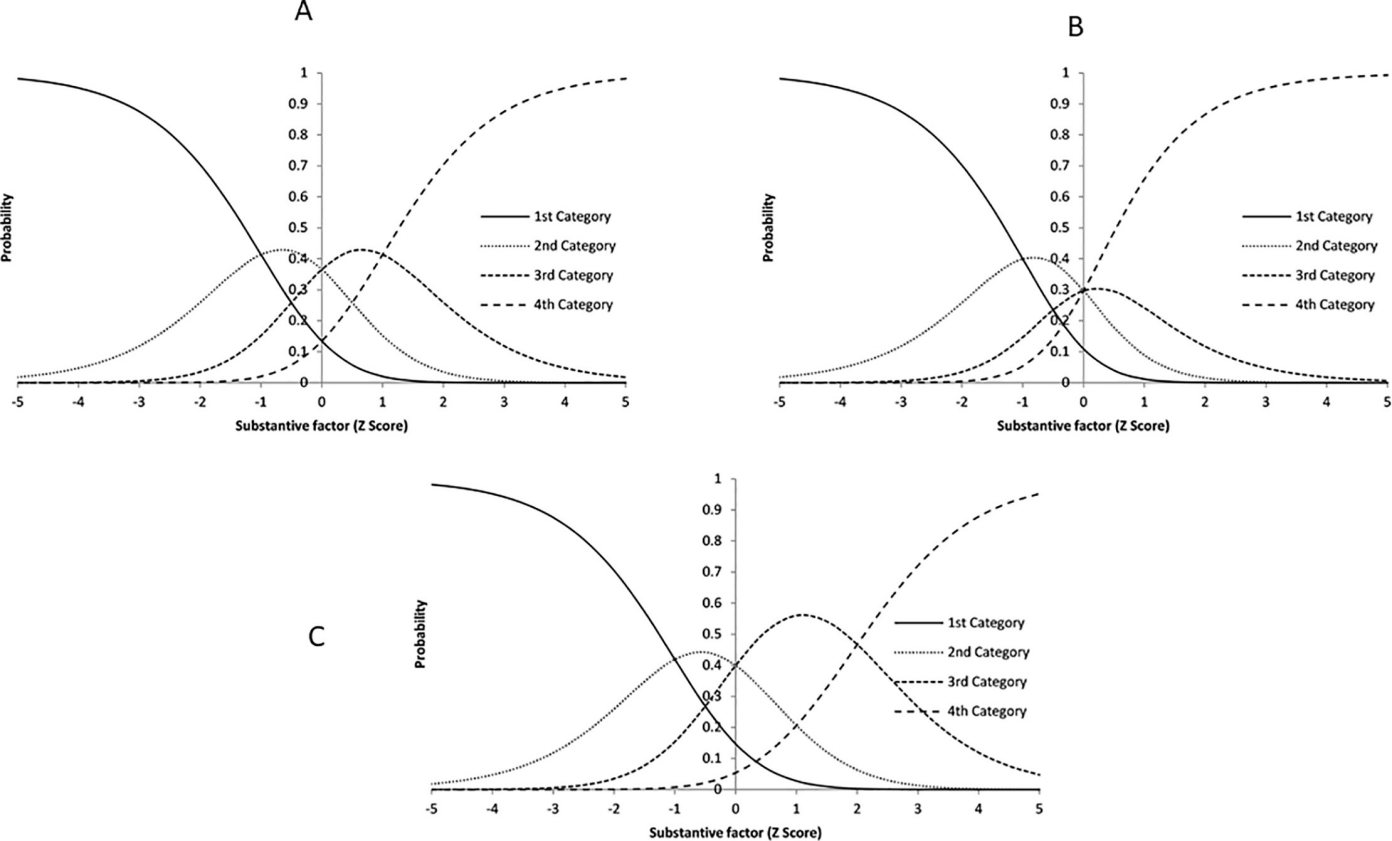
Example of response category curves with different PERS levels. (A) Category Characteristic Curves without PERS bias (*θ*_*PERS*_ = 0). (B) Category Characteristic Curves with positive PERS bias (*θ*_*PERS*_ = 1). (C) Category Characteristic Curves with negative PERS bias (*θ*_*PERS*_ = −1).

In **Fig 1A**, the subjects with scores greater than one have the highest probability of selecting the 4^th^ (highest) category; in **Fig 1B**, the 4^th^ category curve moves to the left, and the score threshold for selecting the 4^th^ category with highest probability decreases to 0, which means more subjects will select this category; in **Fig 1C**, the 4^th^ category curve shifts to the right, and the score threshold for selecting the 4^th^ category increases to 2, which means fewer subjects will select the 4^th^ category, and more subjects will select the 3^rd^ category.

In this model, PERS can be understood as a random effect on the threshold parameter between the 4^th^ category and 3^rd^ category (*c*_*j*,4_).[14] This threshold parameter varies across subjects, that changes the difficulty level of endosing the 4^th^ category for each subject. In addition to variation of the threshold parameter across subjects, in **Formula 1**, the slope of PERS (*a*_*j*,*PERS*_) differs between items, so the impact of PERS varies across items.

#### PERS bias impact

To estimate the impact that PERS bias has on scores, we applied the expected a posteriori method to estimate the person scores based on the two models (with and without adjusted PERS). In each scale, we calculated the correlation between scale scores in the two models and examined scatter plots of the score distributions. First, we calculated the 2.5^th^, 25^th^, 75^th^ and 97.5^th^ percentiles of the PERS factor score distribution. Then at each PERS factor score percentile level, we applied **Formula 2** to calculate the expected summed score at each substantive factor score from -3 to 3 in increments of 0.1. We used the expected summed score calculated for PERS at 0 as the reference, and the bias was calculated as *BIAS*(*θ*_*i*_,*θ*_*PERS*_) = *E*(*θ*_*i*_,*θ*_*PERS*_)−*E*(*θ*_*i*_,0).

**Formula 2.** Formula for calculating the Expected Summed Score

$$E(\theta_{i},\theta_{PERS})=\sum_{j=1}^{M}\sum_{k=1}^{K_{j}}kP(U_{j}=k|\theta_{i},\theta_{PERS}),(i=1,2,3)$$

Where M is the total number of items in each scale, for example in the Family & Friends scale, M = 23; *K*_*j*_ is the total number of categories in item j; *P*(*U*_*j*_ = *k*|*θ*_*i*_,*θ*_*PERS*_) is defined as in formula 1.

To consider the distributions of both the substantive factor and the PERS bias, we also calculated the root mean squared bias (RMSB) as: $RMSB(\theta_{i},)=\sqrt{\frac{\sum_{p=1}^{N}{\left[E(\theta_{p,i},\theta_{p,PERS})-E(\theta_{p},0)\right]}^{2}}{n}}$, where *θ*_*p*,*i*_ and *θ*_*p*,*PERS*_ are the actual observed substantive and PERS factor scores for subject p, *N* is the number of subjects in the sample.

#### Identify related characteristics to PERS

It is of particular interest whether individuals with certain demographic characteristics are more or less likely to display higher PERS factor score but lower substantive factor score. Their PERS bias inflates the summed score in the unadjusted model, which could mask their need for interventions or services. To examine the demographic and clinical factors of those subjects, we established four groups based on subjects with either lower or higher substantive factor score (<0) and higher or lower PERS factor score (>0). We selected 0 as the cut point, because both factor scores are z scores, and 0 is the mean of the scale. We then applied a logistic regression model to examine the significant demographic variables that could predict the group with lower substantive factor score and higher PERS factor score. Variables were selected based upon demographics that have been shown to be related to PERS in the literature generally, and specifically those clinically important in the burn literature. We standardized the continuous independent variables (age and time since burn) into the mean and standard deviation equal to 0 and 1. We reported the odds ratio with a 95% confidence interval (CI).

## Results and discussion

### Data preparation

This analysis included the Family & Friends, Social Interactions, and Social Activities scales with 23, 24, and 14 items, respectively. Each item has 5 response categories. There are two types of response options: agreement from “Strongly Disagree” to “Strongly Agree”, frequency from “Never” to “Always” and from “Not at All” to “A Lot”. We reverse coded 10, 22 and 9 items in the Family & Friends, Social Interaction and Social Activity scales respectively, so the higher response score means higher function level or less impairment. For categories with sample sizes less than 10, we merged responses with the adjacent category (2 items each in Family & Friends and Social Interaction scales, 4 items in the Social Activity scale). The response option content and the sample sizes are listed in S2 Table. We calculated the percentage of subjects selecting the positive extreme response category for each item. An average of 44.89%, 41.47% and 49.30% of subjects selected the positive extreme response option in the Family & Friends, Social Interaction, and Social Activity scales, respectively.

### Building the IRT model to adjust the PERS factor

**Table 1** shows the fit indices for both unadjusted and adjusted by PERS MGPCM. Compared with MGPCM, the one including the PERS factor yields the smaller AIC, BIC and sample-size adjusted BIC, indicating that this model has the better fit.

**Table 1:** Model comparison.

	MGPCM	MGPCM adjusted by PERS
**# of parameters**	299	360
**Loglikelihood**	-36260.23	-35291.66
**AIC**	73118.47	71303.32
**BIC**	74433.65	72886.81
**Sample-Size Adjusted BIC**	73484.40	71743.91

MGPCM: Multidimensional Generalized Practical Credit Model; AIC: Akaike information criterion; BIC: Bayesian information criterion

The correlations between the Family & Friends with Social Interaction, Family & Friends with Social Activity, and Social Interaction with Social Activity scales before adjusting for the PERS factor are 0.70, 0.69 and 0.80, respectively; after adjusting for the PERS factor, the correlations changed to 0.63, 0.70 and 0.83, respectively. PERS factor has the larger effect on the relationship between Family & Friends with Social Interaction where the other two correlations were with much smaller changes. Without considering the PERS factor, the correlation between Family & Friends and Social Interaction scales is over-estimated because of the high percentage of participants who responded to both scales at positive extreme categories; after adjusted PERS, the real correlation is decreasing.

#### PERS bias impact

To assess the impact of PERS bias on LIBRE Profile scores, we examined the correlations between person scores based upon the two different models. Lower correlations indicate a greater difference between the scores generated by the two models, suggesting a greater effect of PERS on scores. S1 Fig displays scatter plots of the person scores based upon the two models. The correlations range from 0.79 to 0.89. In general, the discrepancy between the two scores occurs at the higher end of the scale.

**Fig 2:**
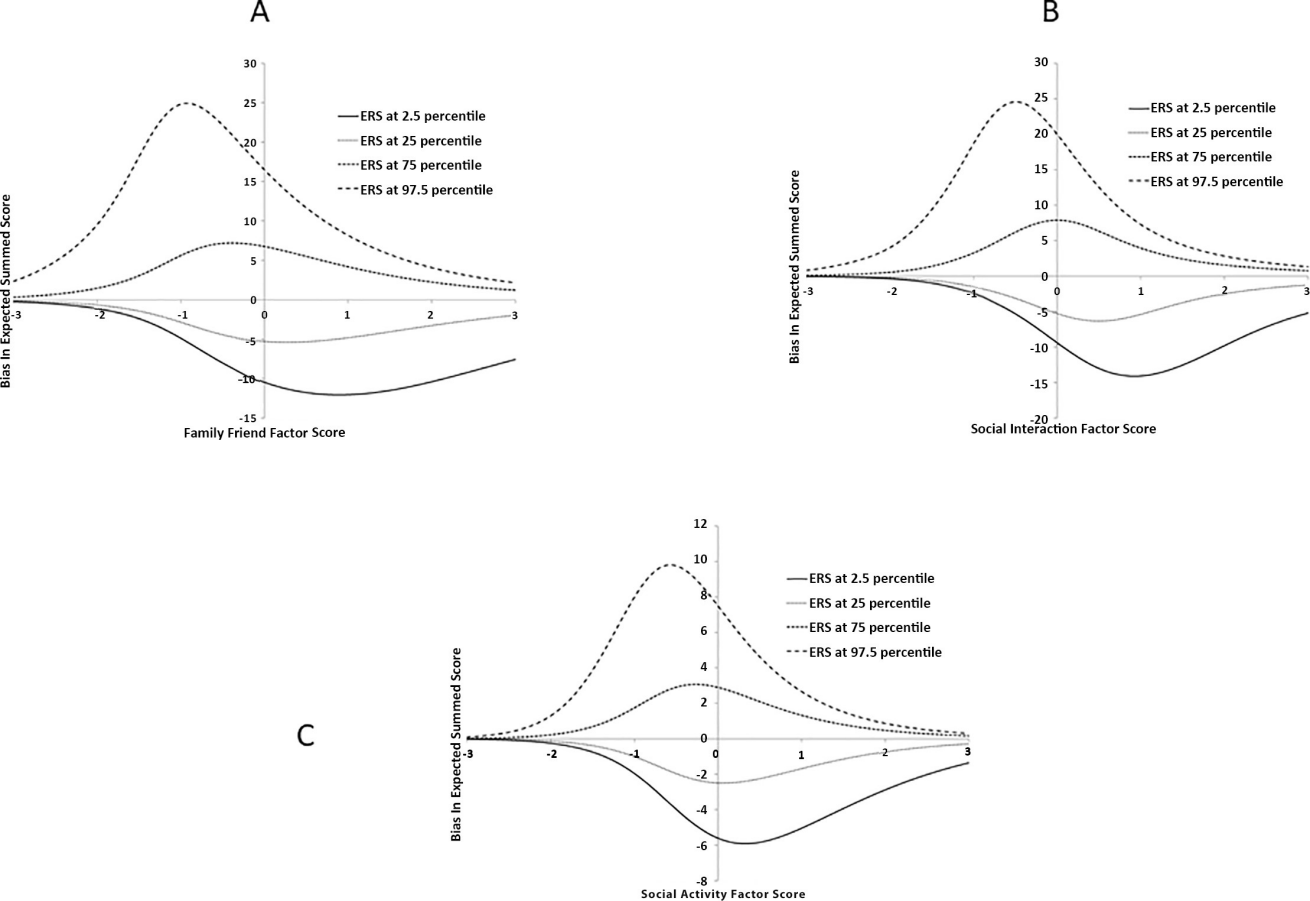
The expected summed score bias at difference PERS levels across the substantive factor score range. Family Friend Factor. (B) Social Interaction Factor. (C) Social Activity Factor.

To examine the impact of PERS, we calculated LIBRE Profile expected summed score bias for individuals who show PERS biases in the 2.5^th^, 25^th^, 75^th^, and 97.5^th^ percentiles (PERS bias of -1.49, -0.54, 0.57 and 1.72, respectively) and those PERS scores at 0 (**Fig 2**). Because the extent of the bias’s impact on the expected summed score may differ depending upon a respondent’s actual ability, at each percentile level of bias examined, we checked the impact of that bias on the expected summed scores across the range of substantive factor scores.

The figures show how PERS can influence LIBRE Profile expected summed scores as either smaller (the lines below the x-axis) or larger (the lines above the x-axis). For individuals with a PERS bias in the 2.5^th^ and 25^th^ percentiles, the expected summed scores would be lower than those with a PERS of 0. The largest bias in this direction occurs around 1 along the substantive factor score. The expected summed scores of Individual with a bias in the 2.5th percentile are underestimated by a magnitude range from -6 in the Social Activity scale to as much as -15 in the Social Interaction scale. However, when respondents’ biases are at the 75^th^ and 97.5^th^ percentiles, the expected summed scores considering the bias would be higher than those with a PERS of 0. The largest bias in this direction happens around -1 along the substantive factor score. Individuals with a bias in the 97.5^th^ percentile have their scale scores overestimated by a magnitude ranging from 10 in the Social Acivity scale to as much as 25 in the Social Interaction and Family & Friends Scale.

S1 Table illustrates response patterns for individual subjects with different extreme response bias scores as examples, and how those PERS biases impact LIBRE Profile scores in the unadjusted model. For case 1773, the PERS score was close to 0, so the three scale scores are similar in both models. For case 1386, the subject responded “0” to all items, clearly showing a preference to select the highest response category. Therefore, the estimated PERS score for this subject was high (1.67), and in the model that does not adjust for the PERS bias, the substantive factor scores are over-estimated. For case 1458, the subject never selected “0” for any item and this indicated that the the subject might prefer not to (or actively avoid) selecting the highest response category, and thus the true scale scores are higher once we adjusted for PERS.

The root mean squared bias (RMSB) is an indicator of the impact of this measurement error; in this case it indicates the average extent the PERS bias has overall on the average scores of this study sample. The RMSB for Family & Friends, Social Interactions, and Social Activity scales are 7.02, 6.77 and 2.83 points respectively. The standard deviations of the expected summed score for the model that includes the PERS were 14.14, 19.60 and 10.41, respectively. Therefore, in standard deviation units, the Social Activity scale has relatively smaller RMSB, 0.27 of one SD, the Social Interaction scale has relatively medium RMSB, 0.35 of one SD, the Family & Friends scale has larger RMSB, 0.50 of one SD. 0.27 to 0.5 SD corresponds from a small to a medium effect size [15].

We also evaluated whether the fit indices of GPCM adjusted PERS model were less than the values in the GPCM model under the situation that a high percentage of persons has higher function level/or positive attitude in a simulation study. The GPCM adjusted PERS model didn’t fit better than the GPCM model in that situation. This finding validated our contention that the positive extreme responses were due to a factor different from the true sentiment of the respondent. We also found that PERS was evident across scales and further supports our contention that PERS is a unique factor rather than the result of respondents’ in what might be their true sentiments (S2 Appendix).

### Identifying related characteristics

Table 2 denotes two groups, the first group contains three types of subjects: (a) the subjects who have both higher scale scores and PERS bias, (b) the subjects who have both lower scale scores and PERS bias, and (c) the subjects who have higher scale scores, but lower PERS bias. The second group, of particular interest, contains the subjects who have lower scale scores, but higher PERS bias. The results from the logistic regression of demographic factors predicting subjects in Group 2 compared with the reference group 1 representing the three types of subjects combined are given in Table 3. Results in bold represent significantly greater odds for group 2 while the italicized results are significantly lower odds of being in group 2.

**Table 2:** Subjects grouped by substantive factor and PERS.

Group	Family & Friends	Social Interaction	Social Activity
1a. Higher substantive factor, higher PERS	171(28.45%)	184(30.62%)	164(27.33%)
1b. Lower substantive factor, lower PERS	110(18.3%)	136(22.63%)	132(22%)
1c. Higher substantive factor, lower PERS	176(29.28%)	150(24.96%)	154(25.67%)
2. Lower substantive factor, higher PERS	144(23.96%)	131(21.8%)	150(24.96%)

PERS: Positive extreme response style

**Table 3:** Logistic regression model predicting subjects in higher PERS factor score and lower substantive factor score group. (Odds ratio and 95% confidence intervals).

Characteristic	Family & Friends	Social Interaction	Social Activity
Male	1.56 (1.02–2.34)*	0.76 (0.48–1.19)	1.09 (0.71–1.67)
White	0.78 (0.47–1.32)	1.23 (0.7–2.15)	1.21 (0.71–2.08)
Age^╪^	*0*.*68 (0*.*53–0*.88)*	*0*.*73 (0*.*56–0*.95)*	0.92 (0.72–1.17)
Time Since Burn^╪^	1.33 (1.04–1.7)*	0.87 (0.66–1.14)	*0*.*67 (0*.*51–0*.88)*
Support Group Participation	0.73 (0.50–1.12)	*0*.*63 (0*.*40–0*.97)*	0.66 (0.43–1.01)
High School Diploma or more	0.84 (0.54–1.29)	0.82 (0.52–1.28)	*0*.*63 (0*.*41–0*.96)*
TBSA	0.88 (0.70–1.11)	1.16 (0.92–1.48)	1.31 (1.04–1.65)*
Genital Burn	2.11 (0.95–4.68)	1.32 (0.56–3.12)	1.32 (0.59–2.97)
Foot Burn	0.97 (0.58–1.63)	1.20 (0.72–2.01)	1.16 (0.71–1.89)
Face Burn	1.65 (1.03–2.63)*	1.82 (1.12–2.96)*	1.45 (0.92–2.28)
Hand Burn	0.76 (0.48–1.21)	0.81 (0.50–1.32)	0.98 (0.62–1.56)
Married	0.67 (0.42–1.09)	1.05 (0.64–1.70)	1.01 (0.64–1.61)
Self Administer (vs. Phone)	*0*.*49 (0*.*28–0*.87)*	*0*.*51 (0*.*28–0*.90)*	*0*.*51 (0*.*30–0*.88)*

^╪^: standardized variable (the standard deviation of original age variable is 15.99 years, the standard deviation of original time since burn variable is 16.18 years)

*: statistically significant (p<0.05)

PERS: Positive extreme response style

For the Family & Friends scale, burn survivors who were male, were younger, had a longer duration since burn injury or a face burn were more likely to be in Group 2. Compared with subjects who selected the phone interview mode, the subjects who selected self-administration were less likely to be in Group 2. For the Social Interaction scale, burn survivors who were older, had participated in a support group, and selected the self-administration mode were less likely to be in Group 2. However, subjects with a face burn were more likely to be in Group 2, compared to those without a face burn. For the Social Activity scale, burn survivors who had a longer time since burn injury, had a higher education level (high school or above), and selected the self-administration mode were less likely to be in Group 2. Subjects with larger total body surface area (TBSA) burned were more likely to be in Group 2, compared to those with smaller TBSA burned.

## Discussion

Burn survivors displayed PERS bias on three measures of social integration/participation that included the LIBRE Profile Family and Friends, Social Interactions, and Social Activities scales. The average impact of this bias, based upon RMSB, ranged from 0.27 to 0.50 of one standard deviation of the scale score.

We identified the demographc risk profiles of the individuals who were more likely to have high PERS masking low substantive factor scores. Clinically, those individuals might need more attention in recoverying from the burn and re-intergrating into the society. The risk profiles were slightly different across the three scales. For the Family & Friends scale, the risk profile was male individuals at a young age, further out in time from the burn injury, with a face burn and using the phone administered mode of administration. In the Social Interaction scale, the risk profile was young individuals, not participating in support groups, with face burn and using the phone administered mode. In the Social Activity scale, the risk profile was individuals, with recent burns, a large TBSA, with less than a high school diploma and using the phone administration mode. To reduce the bias for those individuals who fit those risk profiles, we could switch to the self-administration mode and encourage individuals to participate in social support groups. Another way to decrease the bias is to collect the same information from multiple informants (such as spouses, friends etc.) and fitting to a tri-factor model [16]. The tri-factor model has been considered a psychometrically sound model which could extract the substantive factor across different informants and decrease the subjective bias [17].

Response style biases may be explained by factors that are inherent to the survey taker, such as gender and cultural background, or to components of the survey itself, such as layout or formatting of items, or perhaps a combination of these.[18] There are inconsistent findings regarding demographic traits associated with PERS; some studies show that men tend toward PERS whereas other studies found no gender differences;[7,13] some found PERS bias increases with age, while other studies found no association.[4] We found associations between PERS and gender or education in one of the scales (Family & Friends and Social Activity respectively). However, PERS was less common for older participants in two of the scales (Family & Friends and Social Interaction).

There are several potential explanations for the PERS biases observed in this study, including social desirability or impression management, inattentive survey completion, or unconscious preferences. For all three scales, individuals who self-selected phone administration were more likely to display PERS. This is not necessarily a mode effect, since respondents were given the *choice* of a self-administered questionnaire or a telephone interview. This could be evidence of social desirability bias. This phenomenon has been noted in the literature. Individuals interviewed in person tend toward PERS compared to those who respond to online self-administered surveys.[19] This also might be evidence of selection bias—where there are unmeasured variables about individuals who chose that mode that make them more likely to choose the extreme response option.

Survey format, such as the label, orientation, and ordering of response options may also impact PERS [18,20,21,22,23,24]. The LIBRE Profile response options were purposefully presented vertically. All are labeled with words and display a middle option. One study found that PERS is present if response options range from positive to negative, but also when response options range from negative to positive[25]. The LIBRE Profile arranges the response options negative to positive, but also has reverse coded items. Therefore, the layout of the response options in the LIBRE Profile may have less of an influence on the PERS bias.

Two examples provide clinical scenarios of the applications of these corrections. The first example is of a burn survivor, 35 year old non-white female not married, with more than a high school education, with support group involvement. The clinical characteristics for this individual with burns included: TBSA 20~40% with face and hand burns and 1.75 years from the time of the burn. The adjusted and non-adjusted substantive scores for Family & Friends, Social Interactions and Social Activities were 0.25/1.91, -0.11/1.37, -0.11/1.27, respectively; Another example is a 46 year old, white male, married, with more than a high school education, with support group involvement. The clinical characteristics for this individual with burns included, TBSA 20~40% with face burns and the individual was 25.42 years since the burn injury. The adjusted and non-adjusted substantive scores for Family & Friends, Social Interactions, and Social Activities were 0.53/1.58, -0.36/0.97, 0.24/1.34, respectively. In both cases, the non-adjusted scores were over-estimated by one standard devation compared with the adjusted scores.

There are several limitations to this study. First, because the data set is a convenience sample, we cannot comment on the generalizability of these findings to all burn survivors, or to other clinical or general groups. However, the convenience sample was heterogeneous in terms of a breadth of socio-demographic and clinical characteristics of those that were sampled. Future studies that use the LIBRE Profile in more representative populations or different clinical subsamples should examine PERS in these future samples. Another limitation is that this study did not have any measure of social desirability, which we hypothesize PERS might be associated with. In a future study, we can administer the LIBRE Profile with a social desirability scale and examine the relationship between the PERS factor and the social desirability factor. Another limitation is that it was not feasible to analyze ERS for the lower response categories given fewer cases. Future studies can evaluate this through other samples. The multidimensional IRT model was used to examine PERS because we observed that almost half of the sample responded to the positive extreme category. If we took into account the measurement error and calculated the ratio between the PERS score and measurement error (the absolute value of this ratio is greater than 1.96 and would indicate the PERS score is statistically significant from 0), and in our sample there were 52.08% (313 out of 601) of respondents whose PERS scores were not significantly different from 0. We could say about half of the sample was without the PERS bias. In future work, we can explore whether there are other response style effects on burn survivor samples using a mixed Rasch model (or mixture IRT)[26,27]. Another limitation is the assumption that PERS was uncorrelated with substantive factors. However, we conducted additional analysis to examine the robustness of our findings when the model allowed for the correlations between the PERS factor and substantive factors. We identified similar significant demographic variables in Table 3 when we considered that the PERS was correlated with substantive factors, and the correlations between PERS and substantive factors ranged from -0.387 to -0.159 which is negligible to low correlations [28]. The results were consistent between the models with and without correlations. Since the uncorrelated assumption has been used by similar studies[4,29], we reported the final results based on the model without correlations. Other limitations included the study design itself, where we only explored a limited number of demographic variables which could impact the PERS. Further, PERS also could be impacted by test design (eg. order of the questions, wording of the questions, response options, etc.), Batchelor & Mias(2016)’s meta-analysis of extreme response style indicated that ERS might also be related to race, gender and acquiescence as well [30].

## Conclusions

To conclude, burn survivors are a clinically unique group of individuals. After finding evidence for a unique pattern of PERS in this group, it is possible that other unique clinical samples are likely to display PERS as well. When using self-reported measures in clinically unique samples, PERS should be estimated to assess if the bias is occurring and the extent to which it may be impacting scores. We examined the demographic characteristics for those with higher PERS scores but lower substantive factor scores. That information might be helpful for clinicians to interpret the scores for those subjects with characteristics that may impact scores. Clinicians might consider such information given that they have the required statistical support to identify respondents whose true scores may be lower than what they are reporting. Clinicians should be aware that PRO scores may be biased. In the case of PERS, this bias may conceal lower true scores for respondents that may impact provision of services when used as a needs assessment. When statistical support to clinicians is available, we recommend applying the multidimensional IRT model to estimate PERS scores and corresponding standard errors, and evaluate the impact of the PERS value by testing whether the PERS score is statistically significantly different from 0 (to do that, we will calculate the ratio between the PERS score and the standard error). This will provide the clinician with information on whether we can use the summary score or not. In the future, work can be done where such information is more easily available to clinicians through automated algorithms that are built where these computations are easily made with interpretative guidelines for their use.

This paper describes an important method for evaluating the extent of PERS and its impact on the interpretation of results for the LIBRE Profile. Future work can consider this effect in this measure and for other PROMs.

## Supporting information

S1 Table Response patterns and score estimations under different models. * Score 0 means higher function or better outcome, the first row is for family friend scale, the second is social interaction, the last row is the social activity scale; all the scores were in Z score units. MGPCM: Multidimensional Generalized Practical Credit Model PERS: Positive Extreme Response Style.
(PDF)

S2 Table The content of the response options, the sample size in each response option and the item content. (PDF)

S1 Fig Scatter plots of person substantive factor scores adjusted by PERS or not. (PDF)

S1 Appendix Demographic and clinical characteristics and study criteria. (DOCX)

S2 Appendix Model comparison within each domain. (DOCX)
