# Supplementary material for: Extreme response style bias in burn survivors
Source: PLoS One. 2019 May 6;14(5):e0215898. doi: 10.1371/journal.pone.0215898 (PMC6502351; doi:10.1371/journal.pone.0215898)
Supplement: S1 Table — * Score 0 means higher function or better outcome, the first row is for family friend scale, the second is social interaction, the last row is the social activity scale; all the scores were in Z score units. MGPCM: Multidimensional Generalized Practical Credit Model PERS: Positive Extreme Response Style. (PDF) [file pone.0215898.s001.pdf]

S1 Table. **Response patterns and score estimations under different models:**

		GPCM adjusted by PERS				GPCM		
Subject ID	Response Pattern*	Family & Friends	Social Interactions	Social Activities	PERS	Family & Friends	Social Interactions	Social Activities
1773	00001101110100001100<br>021200122212202111010<br>21002141332213211201	0.34	-0.18	-0.73	0.02	0.36	-0.15	-0.94
1386	000000000000000000000<br>000000000000000000000<br>0000000000000000000	1.51	1.35	1.38	1.67	2.34	2.40	2.65
1458	12121212221112112322<br>32311212211221212321<br>212223122232112111123	-0.28	-0.32	-0.65	-1.97	-0.97	-0.59	-0.84
