# Supplementary material for: Extreme response style bias in burn survivors
Source: PLoS One. 2019 May 6;14(5):e0215898. doi: 10.1371/journal.pone.0215898 (PMC6502351; doi:10.1371/journal.pone.0215898)
Supplement: S2 Table — (PDF) [file pone.0215898.s002.pdf]

S2 Table. The content of the response options, the sample size in each response option and the item content:

Family & Friends Scale

Response Score 1	Response Score 2	Response Score 3	Response Score 4	Response Score 5	Item Content
Strongly Agree (16)	Agree (44)	Neither Agree nor Disagree (65)	Disagree (147)	Strongly Disagree (320)	I don't get along with my family.
Strongly Agree(16)	Agree(54)	Neither Agree nor Disagree(60)	Disagree(171)	Strongly Disagree(284)	Changes in the way I look have interfered with my family relationships.
Strongly Agree(22)	Agree(49)	Neither Agree nor Disagree(53)	Disagree(144)	Strongly Disagree(330)	I have little contact with members of my family.
Strongly Agree,Agree(32)	Neither Agree nor Disagree(45)	Disagree(119)	Strongly Disagree(394)		My family would be better off without me.
Strongly Agree(13)	Agree(27)	Neither Agree nor Disagree(74)	Disagree(199)	Strongly Disagree(266)	I don't like the way most family members act around me.
A Lot(23)	Quite a Bit(19)	Somewhat(57)	A Little Bit(83)	Not at All(396)	I am disappointed in my ability to socialize with my family.
Always(17)	Often(21)	Sometimes(70)	Almost Never(124)	Never(351)	As much as possible, I avoid members of my family.
Always(24)	Often(42)	Sometimes(169)	Almost Never(124)	Never(228)	I would rather be alone than with my family.
Always,Often(19)	Sometimes(79)	Almost Never(172)	Never(314)		I am bothered by the way my friends treat me.
Strongly Agree(17)	Agree(42)	Neither Agree nor Disagree(94)	Disagree(182)	Strongly Disagree(260)	I have trouble keeping friends.
Strongly Disagree(16)	Disagree(28)	Neither Agree nor Disagree(105)	Agree(257)	Strongly Agree(169)	Members of my family enjoy meeting my friends.
Strongly Disagree(25)	Disagree(56)	Neither Agree nor Disagree(52)	Agree(206)	Strongly Agree(254)	There is at least one person in my family that really understands me.
Strongly Disagree(13)	Disagree(19)	Neither Agree nor Disagree(32)	Agree(218)	Strongly Agree(311)	Most family members are comfortable being with me.
Strongly Disagree(26)	Disagree(36)	Neither Agree nor Disagree(76)	Agree(198)	Strongly Agree(258)	Members of my family give me the support that I need.
Strongly Disagree(39)	Disagree(55)	Neither Agree nor Disagree(78)	Agree(209)	Strongly Agree(211)	My family is comfortable talking about burns.
Strongly Disagree(17)	Disagree(24)	Neither Agree nor Disagree(80)	Agree(206)	Strongly Agree(240)	My family is comfortable with my burns being seen in public.
Strongly Disagree(17)	Disagree(53)	Neither Agree nor Disagree(79)	Agree(251)	Strongly Agree(167)	I am comfortable being helped by my family.
Never(12)	Almost Never(42)	Sometimes(121)	Often(195)	Always(210)	I get plenty of help and support from friends.
Not at All(20)	A Little Bit(59)	Somewhat(120)	Quite a Bit(156)	A Lot(234)	I allow myself to become close to my friends.
Strongly Disagree(12)	Disagree(14)	Neither Agree nor Disagree(24)	Agree(167)	Strongly Agree(382)	I have at least one real friend.
Strongly Disagree(43)	Disagree(99)	Neither Agree nor Disagree(78)	Agree(213)	Strongly Agree(164)	I have many friends in the city where I live.
Strongly Disagree(18)	Disagree(41)	Neither Agree nor Disagree(100)	Agree(235)	Strongly Agree(142)	My friends have helped me get out of the house.
Strongly Disagree(33)	Disagree(48)	Neither Agree nor Disagree(118)	Agree(167)	Strongly Agree(141)	I get the support I need from other burn survivors.

Social Interaction

Response Score 1	Response Score 2	Response Score 3	Response Score 4	Response Score 5	Item Content

ERS Bias in Burn Survivors

Always(19)	Often(45)	Sometimes(179)	Almost Never(133)	Never(221)	I limit my activities because of how my burns look.
Always,Often(46)	Sometimes(141)	Almost Never(127)	Never(272)		I avoid going to community events.
Strongly Agree(21)	Agree(52)	Neither Agree nor Disagree(79)	Disagree(150)	Strongly Disagree(280)	I do not go to parties because of how my burns look.
Strongly Agree(29)	Agree(64)	Neither Agree nor Disagree(48)	Disagree(176)	Strongly Disagree(237)	I don't play sports because of how my burns look.
Always(41)	Often(65)	Sometimes(161)	Almost Never(150)	Never(179)	Because of my burns, I am uncomfortable around strangers.
Always(42)	Often(46)	Sometimes(146)	Almost Never(117)	Never(167)	I am upset when strangers avoid looking at me.
Always(42)	Often(46)	Sometimes(157)	Almost Never(122)	Never(221)	I feel uncomfortable in crowds because of my burns.
Always(52)	Often(59)	Sometimes(142)	Almost Never(128)	Never(217)	I feel embarrassed about my burns.
Always(43)	Often(48)	Sometimes(154)	Almost Never(147)	Never(194)	Because of how my burns look, I am uncomfortable when I meet new people.
Always(60)	Often(99)	Sometimes(129)	Almost Never(115)	Never(194)	I avoid doing things that might call attention to my burns.
Always(20)	Often(45)	Sometimes(99)	Almost Never(145)	Never(280)	Because of my burns, I avoid strangers.
Always(37)	Often(67)	Sometimes(166)	Almost Never(122)	Never(202)	I feel like I don't fit in with other people.
Always(22)	Often(28)	Sometimes(96)	Almost Never(133)	Never(316)	I limit going out in public because of my burns.
Always(26)	Often(48)	Sometimes(165)	Almost Never(140)	Never(218)	Because of my burns, I feel uncomfortable in social situations.
Strongly Agree(40)	Agree(83)	Neither Agree nor Disagree(146)	Disagree(188)	Strongly Disagree(126)	I am upset when strangers comment on my burns.
Strongly Agree,Agree(41)	Neither Agree nor Disagree(67)	Disagree(188)	Strongly Disagree(300)		I avoid making new friends because I do not want to talk about my burns.
Always(10)	Often(33)	Sometimes(112)	Almost Never(139)	Never(297)	I avoid being with others because of my burns.
Always(14)	Often(37)	Sometimes(95)	Almost Never(138)	Never(304)	I find it difficult to invite a friend to do something together.
A Lot(22)	Quite a Bit(26)	Somewhat(62)	A Little Bit(110)	Not at All(370)	I am uncomfortable going out with friends.
A Lot(10)	Quite a Bit(19)	Somewhat(49)	A Little Bit(116)	Not at All(395)	I am uncomfortable with friends because of my burns.
Strongly Agree(25)	Agree(53)	Neither Agree nor Disagree(84)	Disagree(173)	Strongly Disagree(255)	Because of my burns, it is hard for me to make friends.
Strongly Agree(14)	Agree(26)	Neither Agree nor Disagree(78)	Disagree(214)	Strongly Disagree(234)	My friends are uncomfortable with me showing my burns in public.
Strongly Disagree(34)	Disagree(121)	Neither Agree nor Disagree(105)	Agree(180)	Strongly Agree(160)	I don't worry about other people's attitudes towards me.
Strongly Disagree(11)	Disagree(19)	Neither Agree nor Disagree(76)	Agree(288)	Strongly Agree(196)	I can help strangers feel comfortable around me.

Social Activity

Response Score 1	Response Score 2	Response Score 3	Response Score 4	Response Score 5	Item Content
A Lot(31)	Quite a Bit(54)	Somewhat(115)	A Little Bit(180)	Not at All(219)	My burns limit me being active.
A Lot(32)	Quite a Bit(46)	Somewhat(104)	A Little Bit(138)	Not at All(275)	/ I am disappointed in my ability / to do leisure activities.
Strongly Agree(51)	Agree(130)	Neither Agree nor Disagree(78)	Disagree(164)	Strongly Disagree(175)	I avoid outdoor activities because of my burns.
Strongly Agree(48)	Agree(106)	Neither Agree nor Disagree(59)	Disagree(148)	Strongly Disagree(232)	I am limited in what I can do for my family.
A Lot,Quite a Bit(21)	Somewhat(44)	A Little Bit(75)	Not at All(397)		My family is upset that I can't do more things with them.
Always,Often(22)	Sometimes(88)	Almost Never(120)	Never(346)		My burns interrupt simple family activities.

A Lot,Quite a Bit(18)	Somewhat(53)	A Little Bit(107)	Not at All(375)		My friends are disappointed in my ability to do things with them.
A Lot(36)	Quite a Bit(48)	Somewhat(71)	A Little Bit(119)	Not at All(271)	I am upset that my burns limit what I can do with friends.
A Lot(29)	Quite a Bit(57)	Somewhat(59)	A Little Bit(182)	Not at All(256)	I tire easily when I go out with friends.
Never,Almost Never(26)	Sometimes(84)	Often(127)	Always(352)		I am able to go to all the community events that are important to me.
Not at All(19)	A Little Bit(46)	Somewhat(111)	Quite a Bit(210)	A Lot(211)	How much do you enjoy your social life?
Never(14)	Almost Never(22)	Sometimes(99)	Often(137)	Always(311)	I am able to do all of my regular family activities.
Never,Almost Never(19)	Sometimes(107)	Often(152)	Always(318)		I am able to socialize with my friends.
Not at All(30)	A Little Bit(43)	Somewhat(111)	Quite a Bit(158)	A Lot(246)	I am satisfied with my ability to do things for my friends.
