# Supplementary figures and images for: Extreme response style bias in burn survivors
Source: PLoS One. 2019 May 6;14(5):e0215898. doi: 10.1371/journal.pone.0215898 (PMC6502351; doi:10.1371/journal.pone.0215898)

**S1 Figure. Scatter plots of person substantive factor scores adjusted by PERS or not**

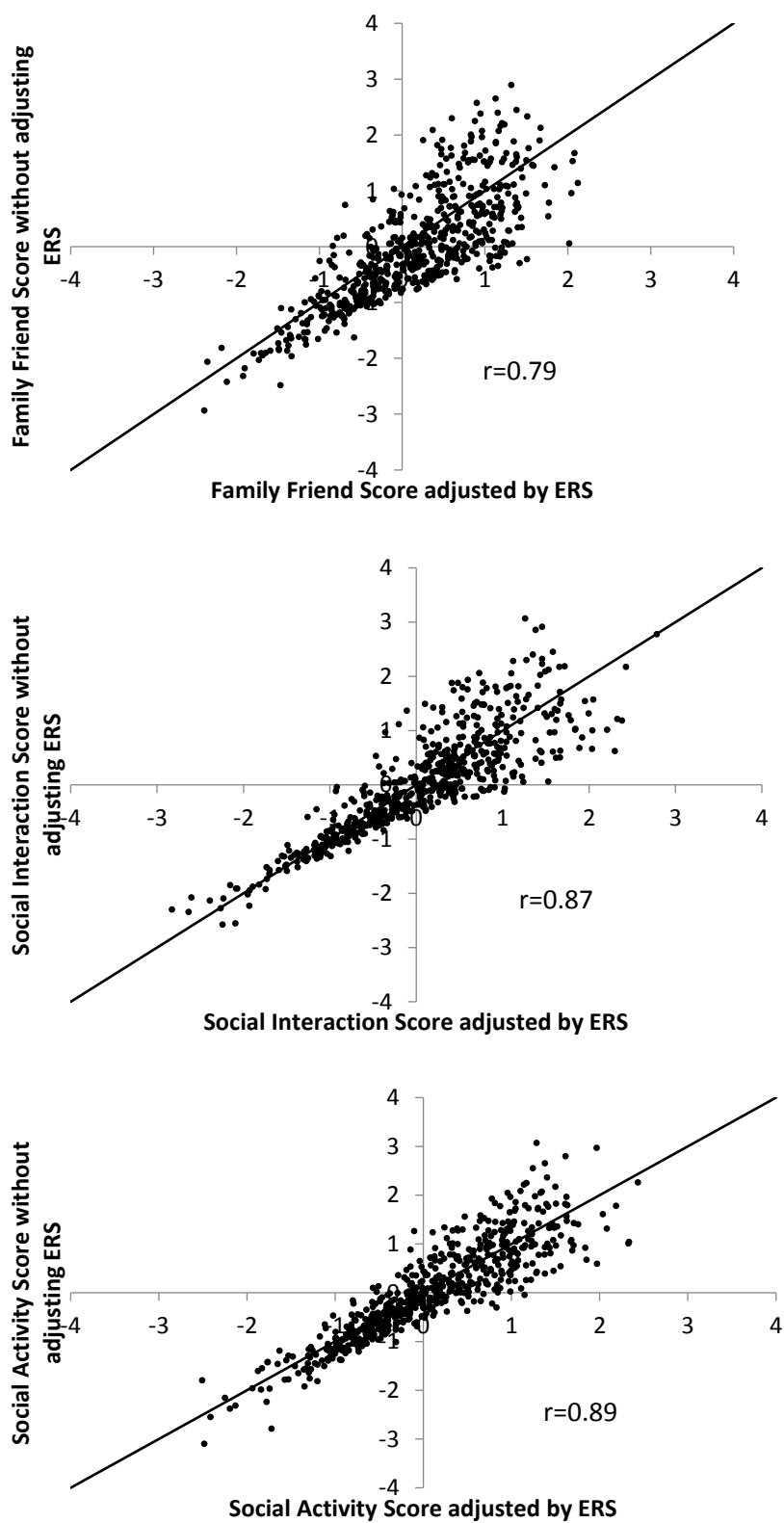

Supplement: S1 Fig — (PDF) [file pone.0215898.s003.pdf]
