# Supplementary material for: Extreme response style bias in burn survivors
Source: PLoS One. 2019 May 6;14(5):e0215898. doi: 10.1371/journal.pone.0215898 (PMC6502351; doi:10.1371/journal.pone.0215898)
Supplement: S1 Appendix — (DOCX) [file pone.0215898.s004.docx]

**Appendix 1. Demographic and Clinical Characteristics and Study Criteria**

The sample included 601 burn survivors;

| Demographic Variables | Mean (SD) or N(%) |
| --- | --- |
| Female | 54.7% |
| Age | 44.6(15.98) |
| Average of total body surface area (TBSA) | 40.5% |
| TBSA<10% | 32.78% |
| TBSA<29% | 13.63% |
| TBSA<49% | 26.29% |
| TBSA>=50% | 27.29% |
| Average of Time Since Burn (years) | 15.4 |

Following criteria were used to select sample:

(1)Age 18 or older, (2) having survived a burn that was at least 5% total body surface area burned (TBSA), and/or burns to one of four critical areas (face, hands, feet, and genitals), (3) living in the United States or Canada, and (4) had the ability to read and understand English.

The main sources of recruitment were the Phoenix Society (a support network for burn survivors), peer support networks, social media, and mailings. Interested persons were screened for eligibility by phone.
