# Supplementary material for: Extreme response style bias in burn survivors
Source: PLoS One. 2019 May 6;14(5):e0215898. doi: 10.1371/journal.pone.0215898 (PMC6502351; doi:10.1371/journal.pone.0215898)
Supplement: S2 Appendix — (DOCX) [file pone.0215898.s005.docx]

**Appendix 2. Model Comparison within Each Domain**

| Domain | Model | AIC | BIC | Sample-Size Adjusted BIC |
| --- | --- | --- | --- | --- |
| Family & Friend | GPCM | 28613.642 | 29110.495 | 28751.751 |
|  | GPCM adjusted by PERS | 27732.727 | 28330.710 | 27898.947 |
| Social Interaction | GPCM | 28563.411 | 29082.445 | 28707.827 |
|  | GPCM adjusted by PERS | 27749.476 | 28374.077 | 27923.265 |
| Social Activity | GPCM | 16513.728 | 16799.637 | 16593.279 |
|  | GPCM adjusted by PERS | 16291.288 | 16638.777 | 16387.973 |
